# Incident changes in the prevalence of influenza type A virus among children before and after COVID‐19 pandemic in Hangzhou, China

**DOI:** 10.1111/crj.13659

**Published:** 2023-06-29

**Authors:** Xiucui Han, Qing Ye, Pengfei Xu

**Affiliations:** ^1^ Department of Clinical Laboratory, Children's Hospital Zhejiang University School of Medicine, National Clinical Research Center for Child Health, National Children's Regional Medical Center Hangzhou China; ^2^ Clinical Laboratory Zhejiang Hospital Hangzhou China

Dear editor,

The report entitled “Incident changes in the prevalence of influenza virus during COVID‐19 pandemic in Hangzhou, China” by Xu et al aroused our strong concern.^1^ In this report, Xu et al announced that the incidence of influenza viruses during the COVID‐19 epidemic was changed. Here, we want to compare the epidemic trend of influenza type A virus in the period of December to February of the next year when the Chinese government optimized the COVID‐19 epidemic prevention measures (2022–2023) to the same period during the COVID‐19 epidemic (2019–2020, 2020–2021, and 2021–2022).

Influenza is a seasonal infectious virus that usually occurs in winter.^2^, ^3^ Influenza viruses belong to the family of Orthomyxoviridae of RNA viruses.^4^ The influenza virus is an enveloped virus whose genome is consisted of a segmented, single negative‐strand RNA encoding surface glycoproteins of hemagglutinin (HA) and neuraminidase (NA).^5^, ^6^ There are seven genera of influenza virus: Alpha influenza virus (influenza type A virus), Beta influenza virus (influenza type B virus), Gamma influenza virus (influenza type C virus), Delta influenza virus (influenza type D virus), Isa virus, Quaranja virus, and Thogoto virus.^7^, ^8^ Influenza type A virus is the most common influenza infection during the flu season and causes mild to severe illness and affects not only humans but also animals.^9^, ^10^ Influenza type A virus can be classified according to the antigenic variation in HA and NA antigens. So far, 16 antigenic variants of HA and nine antigenic variants of NA have been identified.^4^ In addition, several combinations of HA and NA may also serve as subtypes (e.g., H1N1, H5N1, and H7N9). The diagnosis of influenza is usually based on clinical basis, laboratory tests, epidemiological information, and symptoms of influenza infection. The incubation period of influenza type A virus is usually 1–3 days. The main symptoms are fever, sore muscles, and headache. There are also some mild respiratory symptoms, such as cough, runny nose, sore throat, and so forth.

In this study, we analyzed the epidemic situation of influenza type A virus in children who came to Children's Hospital of Zhejiang University School of Medicine from December to February in 2019–2020, 2020–2021, 2021–2022, and 2022–2023.

At the beginning of December 2022, the Chinese government released the “new ten” optimization measures for the prevention and control of the COVID‐19, and the epidemic was fully liberalized on 7 December 2022. This measure does not mean the end of the epidemic but the optimization of nucleic acid detection and people's travel. Because of the ceasing of comprehensive nucleic acid testing and the relaxation of the prevention and control measures, the country ushered in the peak of COVID‐19 infection. After the peak of infection in the next 1–2 months, the number of infected people becomes more stable and controllable, as well as in children. The infection rate and the number of infected people in Children's Hospital of Zhejiang University School of Medicine showed a linear downward trend (Figure 1). It is worth noting that the infection rate of children in the early stage may be too low, mainly because of the following reasons: On the one hand, because of the lack of medical resources in the early stage, there may be a certain degree of missed detection rate; on the second hand, most children infected are asymptomatic or mild without medical treatment.

**FIGURE 1:**
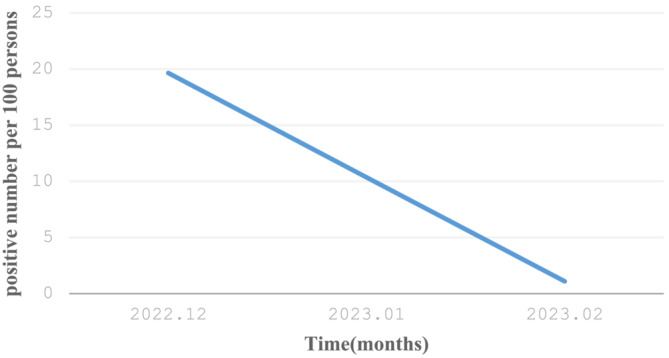
Monthly rate of patients with laboratory‐confirmed COVID‐19 (Hangzhou, China, December to February, 2022–2023).

After the COVID‐19 epidemic situation gradually stabilized, as most people had a 3–6 month protection period after the first infection with COVID‐19, people ignored the wearing of masks and increased interpersonal communication. Compared with the same period during the COVID‐19 epidemic, the incidence rate of influenza type A virus showed a significant upward trend. From the results, we can see that the infection rate of influenza type A virus continued to decline from December 2019 to February 2020, which may be related to the prevention and control measures after the outbreak of the COVID‐19 epidemic. At the same time, in the same period, the peak of influenza type A virus infection rate in 2022–2023 lagged behind that in 2019–2020. The years 2020–2021 and 2021–2022 were in the COVID‐19 prevention and control period, and influenza type A virus was not prevalent (Figure 2). After the COVID‐19 outbreak, the high infection rate of influenza type A virus was related to the low vaccination rate of influenza vaccine during the COVID‐19 epidemic, and the decline of immunity was caused by the long‐term non infection of influenza type A virus in the population.

**FIGURE 2:**
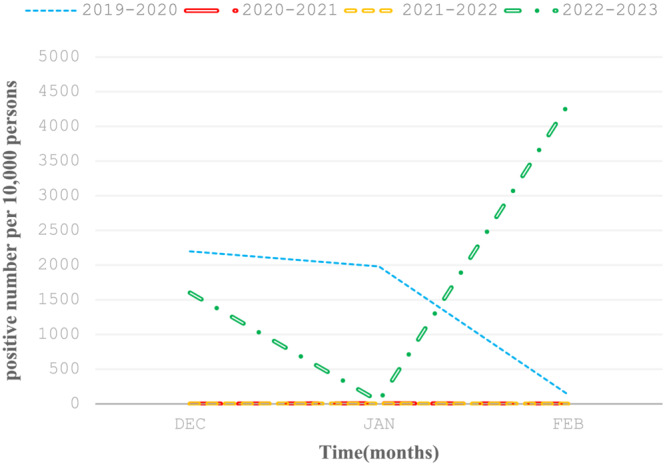
Monthly rate of patients with laboratory‐confirmed influenza type A virus (Hangzhou, China, December to February, 2019–2020, 2020–2021, 2021–2022, and 2022–2023).

In conclusion, the implementation of the “new ten” prevention and control measures for the COVID‐19 has changed the prevalence of influenza type A virus among children in Hangzhou. On the one hand, it is caused by the epidemic characteristics of influenza type A virus itself. On the other hand, under the control of the “dynamic zero” policy during the COVID‐19 period, the wearing of masks and the reduction of social interaction have reduced the incidence rate of influenza type A virus. After 3 years of epidemic, the population's resistance to influenza type A virus has declined. In addition, as a susceptible group of influenza, children's low vaccination rate and weak resistance are also susceptible factors. Although according to the report of Zhejiang Provincial Center for Disease Control and Prevention on 28 February 2023, H1N1 was the main influenza type A virus epidemic strain at present. Because the incidence rate and number of influenza type A virus cases are on the rise, continuous attention and detection will help to predict the epidemic trend, find out whether there are any variation of the virus as soon as possible, and provide a scientific basis for formulating influenza prevention and control strategies. It is worth noting that this study, as a single‐center study, may draw different conclusions in other regions. Specific measures to control respiratory virus infection should be further studied in multiple centers.

In short, during the COVID‐19 pandemic, nonpharmacological interventions reduced the infection rate of children's respiratory viruses. Continuous testing helps prevent a major outbreak of influenza type A virus infection in the period after the COVID‐19 outbreak. We should be alert to the emergence of new variants of influenza viruses and the prevalence of other respiratory viruses.

## AUTHOR CONTRIBUTIONS

Xiucui Han contributed to study design, collected the clinical data, and wrote the manuscript. Qing Ye contributed to the analysis of results and drew the graphs. Pengfei Xu was responsible for the modification and gave the final approval of the manuscript. All authors read and approved the final manuscript.

## FUNDING INFORMATION

This research did not receive any specific grant from funding agencies in the public, commercial, or not‐for‐profit sectors.

## CONFLICT OF INTEREST STATEMENT

All authors have declared that there are no conflicts of interest.

## Data Availability

The data that support the findings of this study are available from the corresponding author upon a reasonable request.
